# Marginal Adaptation and Microleakeage of Directly and Indirectly Made Fiber Reinforced Composite Inlays

**DOI:** 10.2174/1874210601105010033

**Published:** 2011-03-16

**Authors:** Kumbuloglu Ovul, Tezvergil-Mutluay Arzu, Saracoglu Ahmet, Lassila Lippo VJ, Vallittu Pekka K

**Affiliations:** 1 Ege University, School of Dentistry, Department of Prosthodontics, Izmir, Turkey; 2 University of Turku, Institute of Dentistry, Department of Prosthodontics and Turku Clinical Biomaterials Center, Turku, Finland

**Keywords:** Fiber reinforced composite, Indirect technique, Direct technique.

## Abstract

**Aim:**

This study evaluated *in vitro* microleakage of inlays made by direct or indirect techique with or without fiber reinforced composite (FRC) substructure.

**Materials and Methods:**

Standardized mesio-occlusal cavities were prepared and restored using direct-technique with composite resin only or FRC-composite resin, and indirect technique with laboratory composite only or FRC-laboratory composite resin. After thermocycling, teeth were immersed in basic fuchsin dye, sectioned and examined under a stereo-microscope (x40).

**Results:**

No differences of cement thickness and dye penetration were found in gingival area (p>0.05), whereas microleakage revealed statistical differences between groups (p=0.02) in occlusal area, where FRC-groups had lower microleakage than composite restorations. Thickness of cement layer did not show significant difference between groups with indirect technique (p>0.05).

**Conclusion:**

The present study suggests that insertion of FRC substructure to the inlay cavity by direct composite filling technique does not increase the marginal leakage compared to that of cementing indirectly made restotorations by composite resin luting cement.

**Clinical Significance:**

On the basis of the results of this in vitro study, the use of direct  FRC technique might be an effective way to decrease the marginal leakage.

## INTRODUCTION

Fixed partial dentures (FPD) of various kinds have been available as a prosthodontic treatment option. Recently, introduction of metal-free restorative materials has led to the use of ceramic and composite FPDs as an alternative to conventional porcelain-fused-to-metal restorations. An inlay retained metal-free FPD may be feasible option in an esthetic tooth replacement with a minimally invasive reduction of abutment teeth [1-6]. Metal free inlay retained FPDs can be made with durable zirconia or fiber-reinforced composite (FRC) framework [7]. Zirconia restorations have shown limitations in their bonding properties to luting cements [7-9], whereas indirectly made FRC restorations that have semi-interpenetrating polymer network polymer matrix in the substructure and bonding site provide reliable bond to the composite resin luting cements [9-12]. Alternatively, an inlay retained FPD can also be made by direct technique, i.e. directly in patients mouth. They show promising early clinical outcome [13,14] Both directly and in indirectly made FRC inlay-retained FPDs are luted adhesively. In the directly made FPD there is one adhesive interface (between dentine/enamel and restorative material) whereas in the indirectly made FPD there are two adhesive interfaces (between dentine/enamel and luting cement; and luting cement and FPD). It is not known yet, whether there are differences between quality of sealing by the adhesive interface with indirectly and directly made FRC inlay retaining units.  It was hypothesized that there would be no difference in microleakage between tooth and inlays made by direct or indirect techique with and without FRC substructure.   

## MATERIALS AND METHODS

Forty human premolars free of visible caries were used within one month of extraction. Upon collection, adhering soft tissues and blood were removed under running water and the teeth were stored in chloramin-T solution at 5˚C until use. Before preparation, the teeth were mounted in a cylindrical block (diameter: 2.5 cm), 2 mm below the cementoenamel junction (CEJ), using self-cure acrylic resin (Palapress, Heraeus Kulzer, Germany). Standardized mesio-occlusal cavities were prepared, having gingival margins 0.5 mm above the CEJ. All the preparation margins were located inside the enamel, and the completed preparations had a minimal depth of approximately 2 mm at the occlusal part of the cavities.  

The adhesive system, particulate filler composite resin, flowable composite resin and FRC used in this study are listed in Table **1**. All procedures were constructed according to the manufacturer’s instructions. Prepared teeth were randomly assigned to four groups of ten teeth each and restored with inlays as follows:

Group 1 (control): Direct technique with TetricCeram composite resin

Group 2: Indirect technique with Sinfony laboratory composite resin

Group 3: Direct technique with everStick FRC and TetricCeram composite resin

Group 4: Indirect technique with everStick FRC and Sinfony laboratory composite resin. 

For the direct application of composite (Groups 1 and 3) prepared cavities were acid etched with 37% phosphoric acid (Ultra etch, Ultradent products, South Jordan, Utah) for 15s , then rinsed and air-dried. Primer was applied for 15s (Syntac Primer, Ivoclar-Vivadent, Schaan, Liechtenstein) and air-dried. Adhesive was applied for 10s (Syntac Adhesive) and air-dried. Bonding agent was applied and gently air-dried (Heliobond, Ivoclar-Vivadent, Liechtenstein). Layer of flowable composite resin was applied and polymerized for 40s (Tetric Flow, Ivoclar-Vivadent, Schaan, Liechtenstein). In Group 3, following the flowable composite application, a layer of unidirectional resin impregnated FRC (everStick C&B, StickTech, Turku, Finland) was applied to the cavity using a silicon instrument (Refix D, StickTech), and incrementally restored by composite resin.  Fibers of the FRC were oriented mesio-distally as a simulation of the direction of fibers in the main framework of a FRC FPD. The incremental layers were polymerized with a hand light-curing unit (Elipar, ESPE, Seefeld, Germany) for 40 s. The wavelength of the unit was between 380 and 520 nm. Light intensity was 730 mW/cm² measured by a radiometer (Optilux Radiometer Model–100 SDS Kerr, Danbury, CT, USA). 

For the fabrication of indirect restorations (Groups 2 and 4), impressions of the prepared cavities were taken using polyvinylsiloxane impression material (Elite H-D, Zhermack SpA, Badia Polesine, Italy), and cast in vacuum-mixed Type IV dental die stone (Fujirock, GC Corp, Tokyo, Japan). Stone dies were carefully separated from the impressions and two coats of die spacer (Spacer-Tray, Kerr Corporation, West Collins, California) were applied. Inlays of Group 2 received a layer of flowable composite, followed by the laboratory composite application, and inlays of Group 4 received a layer of FRC after the flowable composite onto which laboratory composite was veneered. The initial polymerization of the inlays were made by using a hand light-curing unit (Elipar) for 40 s. In addition to light polymerization, they were further polymerized in a light-curing oven (LicuLite, Dentsply De Trey GmbH, Dreieich, Germany) for 15 min in which the heat rose to ca. 80 C. 

Dual-cure resin luting cement (Variolink II, Ivoclar-Vivadent, Schaan, Liechtenstein) was used for the cementation of the indirectly made restorations. The etching, bonding and priming steps followed the same procedure as in direct restoration group. Before cementation, the luting surfaces of the indirect inlay restorations were roughened with rotating siliconcarbide stone bur and bonding agent (Heliobond) was applied, kept in a dark container for five minutes and gently air-dried. Variolink II luting cement was applied to the inlay surface, then the restoration was seated into the cavity and polymerized for 40s. 

**Fig. (1):**
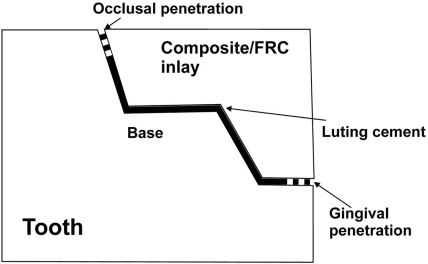
Schematic figure representing cross section of one of the specimens.

For the finishing and polishing of the restoration, flexible discs (Sof-Lex, 3M ESPE) were used on proximal surfaces and fine diamonds on the occlusal surfaces. The specimens were first stored in water at 37°C for 24 h and then subjected to thermocycling in deionized Grade 3 water for 6000 cycles between 5°C and 55°C, with a dwell time of 30 s and a transfer time of 5 s. Twenty-four hours after thermocycling, all tooth surfaces were coated with a nail varnish, except a 1mm-wide zone around the margins of the restoration. After sealing, the teeth were immersed in 0.5% basic fuchsin dye for 24 h at 37°C and finally cleaned in running water before sectioning. The teeth were sectioned by 3-5 sagittal cuts using a diamond cutting saw (Ernst Leitz GMBH, Wetzlar 1600, Germany). The sectioned restorations were examined under a stereo-microscope (Stereomicroscope, Wild M3B, Heerbrugg, Switzerland) at x40 magnification to evaluate the microleakeage and the thickness of the cement layer. The stain depth (mm) from the occlusal or gingival surfaces and cement thickness (mm) from the occlusal, gingival or from the bottom of the cavity were digitally measured (Fig. **1**) (Leica DC Twain, Leica Microsystem Imaging Solutions Ltd). 

The data for all the groups were analysed statistically with SPSS 14.0 (Statistical Package for Statistical Science, SPSS Inc., Chicago, Illinois, USA). The factorial analysis of variance (ANOVA) was used to investigate the main significant effects of location and restorative material on cement thickness and direct *vs* indirect application and margin location on the dye penetration. Further, for interactions, one-way ANOVA and Dunnetts T3 post hoc test were used. The level of significance was set at α=0.05.

## RESULTS

The mean thickness values of the cement layer for indirect FRC and composite inlays are presented in Fig. (**2**). The amount of dye penetration from occlusal and gingival aspects is given in Fig. (**3**). The statistical analysis of the cement thickness among the indirect restoration groups showed no significant difference between the restorative materials (p>0.05), however, significant differences were found among different locations (p<0.05), gingival thickness and occlusal cement thickness being more than the thickness at the cavity base. Factorial ANOVA showed that the restoration technique (direct vs indirect) did not have significant effect (p>0.05), but the restorative material had significant effect on the dye penetration depth (p<0.05). The interactions between the factors were also significant (p<0.05). For interactions, one way ANOVA was performed. Microleakage in gingival area revealed no difference between groups (p>0.05). In occlusal surfaces, groups had significant differences with regard to microleakage (p=0.02), where FRC-groups had a tendency for lower microleakage than composite restorations. Thickness of cement layer did not show significant difference between groups with indirect technique (p>0.05). 

## DISCUSSION

**Fig. (4):**
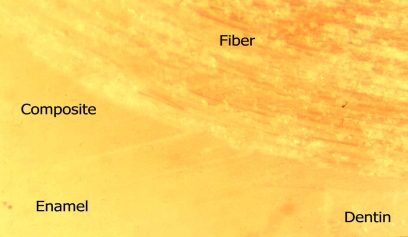
Stereomicroscope illustration (x40) Group 3.

**Fig. (5):**
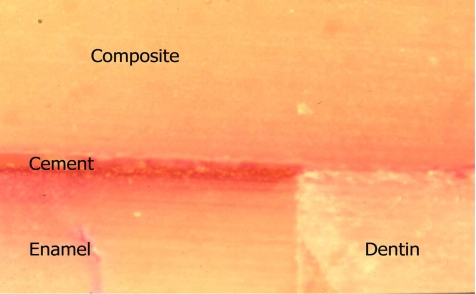
Stereomicroscope illustration (x40) Group 2.

This study was designed to compare marginal sealing of composite resin inlay restorations used as retaining elements of FRC inlay FPDs. The rationale for the comparison came from the existing knowledge and clinical practice of reducing marginal leakage of composite restorations caused by polymerization shrinkage with indirectly made restorations [15-17]. On the other hand, the use of FRC in the inlay retained FPDs by direct technique has proved to be time-saving and clinically successful way of fabricating the FRC FPDs [14]. Therefore, it was of interest to investigate the marginal sealing of restorations made directly versus indirectly, and those containing unidirectional FRC as substructure and those without the substructure. (Figs. **4-5**).

Marginal seal is one of the most important factors for the success of a restoration [18]. An effective bond to enamel and dentin would be one of the key issues to reduce marginal microleakage. Previously, it was shown that bonding of indirectly and directly made FRC restorations to tooth does not differ from each other although there were some differences between the fracture patterns [19, 20]. Many studies have shown that bonding of the restorative material to enamel is adequate to resist polymerization contraction stress whereas, the cervical enamel/dentin and composite resin interface has been reported to be more vulnerable to microleakage [18, 20]. According to the present study, microleakage at the occlusal margin and gingival margins did not show any differences when direct restorations with and without FRC substructure were compared. This was an interesting finding, because it is known that unidirectional FRC is anisotropic in mechanical properties, in thermal expansion, and also with respect to the polymerization shrinkage [17-19]. Along the direction of fibers, practically no shrinkage of the FRC occurs whereas in the perpendicular direction significant shrinkage occurs. In the present study design, the fiber direction was far most optimal in terms of polymerization shrinkage and it was expected that marginal leakage at gingival area would have been highest in the group of directly made restorations with the FRC substructure. Similar unexpected early results have been found with individually formed FRC root canal posts when FRC and composite resin luting cement was polymerized simultaneously in the root canal compared to those where the post was polymerized before cementation (“indirectly made post”) [21]. The cavity configuration factor or C-factor, describes the ratio of bonded to unbonded, or free, surface area [22]. Root canal is a high C-factor cavity, minimal if any gap formation between dentine and post was found. This phenomenon can be related to the formation of polymerization shrinkage stress by the resin system of semi-interpenetrating polymer network polymer, which is plasticized by macromolecules of polymethyl-methacrylate. This requires further investigation. The control of initial light irradiance has been associated to the quality of the marginal seal in composite restorations [23, 24]. To simulate the clinical application light irradiance from the top and sides of the restoration were used in this study.  

In the case of indirectly made restorations, no difference in marginal leakage was found at the gingival area. However, by the occlusal observation, the inlay with the FRC substructure showed significantly less marginal leakage than in other groups. There was no difference in the thickness of the luting cement between indirectly made restorations with and without FRC substructure. The thickness of the cement layer varied between 0.06 and 0.09 millimeters. This shows acceptable fit of the restorations.

From the clinical perspective, the results of the present study show that, directly made inlay retained FRC FPDs which have FRC substructure with fiber direction in mesiodistal direction, i.e. from cavity of one abutment to the cavity of another abutment, are not more prone for marginal leakage than indirecly made restorations. The mesio-distal fiber direction is important for the load-bearing capacity of the FRC FPD: according to the Krenchel´s factor for the reinforcing efficiency on fibers and loading conditions by biting function, mesio-distally placed fibers from cavity to cavity act effectively as occlusal support against vertical occlusal forces [25]. In designing FRC substructure for FRC FPD, other required substructure elements are supports for the pontics and additional surface retained bonding wings in canines [26,27].

## CONCLUSIONS

The present study suggests that insertion of FRC substructure to the inlay cavity by direct composite filling technique does not increase the marginal leakage compared to that of cementing indirectly made restotorations by composite resin luting cement

## CLINICAL SIGNIFICANCE

On the basis of the results of this in vitro study, the use of direct  FRC technique might be an effective way to decrease the marginal leakage.

## Figures and Tables

**Fig. (2):**
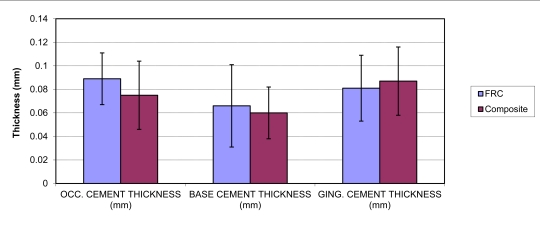
Mean cement thickness (mm) and standard deviations in gingival, occlusal, and cavity base locations. Vertical lines represent standard deviations.

**Fig. (3):**
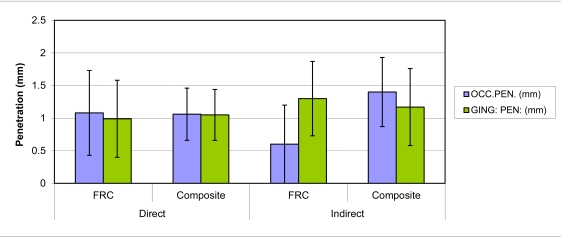
Mean penetration depth (mm) and standard deviations of test groups.

**Table 1:** Materials Used in Study

Product	Type	Manufacturer	Material composition
Tetric Ceram	Composite resin	Ivoclar-Vivadent, Schaan, Liechtenstein	Bis-GMA, UDMA, TEGDMA, Fillers
Tetric Flow	Primer	Ivoclar-Vivadent, Schaan, Liechtenstein	Bis-GMA, UDMA, TEGDMA, Fillers
Heliobond	Resin	Ivoclar-Vivadent, Schaan, Liechtenstein	Bis-GMA, TEGDMA
Syntac	Bonding agent	Ivoclar-Vivadent, Schaan, Liechtenstein	Mixture of water, glutaraldehyde, maleic acid and polyethyleneglycoldimethacrylate
Sinfony	Hybrid composite resin	3M ESPE, St Paul, MN,USA	Dicyclpentyl Dimethylene Diacrylate, Diurethane Dimethacrylate, Fillers
Variolink II	Dual cure resin luting agent	Ivoclar-Vivadent, Schaan, Liechtenstein	Bis-GMA, TEGDMA, UDMA
Everstick	resin-preimpregnated unidirectional FRC	Stick Tech. Ltd., Turku, Finland	PMMA, Bis-GMA, E-glass fibers

bisGMA = bisphenol-A-glycidyl dimethacrylate TEGDMA = triethylene glycol dimethacrylate UDMA = urethane dimethacrylate PMMA = polymethylmethacrylate FRC = fiber-reinforced composite E-glass = electrical glass fibers, silanated
